# Odontomes

**Published:** 1869-01

**Authors:** 


ODONTOMES.—M. Broca, the distinguished surgeon and physiologist, has just elucidated the pathology of the follicles of the teeth, the normal evolution of which had already been described in works on histology. M. Broca does not think that the deviations from this normal evolution give rise to peculiar products, but only to tumors made up of the general hypertrophy of the dental substance These tumors, to which the author gives the name of "odontomes," present two forms : some always remain in the state of more or less soft tumors; whilst others, either wholly or in part, assume the hardness of teeth, producing shapeless, irregular dental masses, sometimes growing to a very large size. In fact, any tumor formed from one or more of the substances entering into the formation of a tooth is due to the dentification of a soft tumor of the same form and volume which originally contained hypertrophied odontogenic tissues. This hypertrophied tumor stands in the same connection, with regard to the dentified tumer, as the normal dental bulb does to the healthy tooth.—*Lancet.*

